# PET Molecular Imaging in Drug Development: The Imaging and Chemistry Perspective

**DOI:** 10.3389/fmed.2022.812270

**Published:** 2022-02-28

**Authors:** Sridhar Goud Nerella, Priti Singh, Tulja Sanam, Chander Singh Digwal

**Affiliations:** ^1^Department of Neuroimaging and Interventional Radiology, National Institute of Mental Health and Neurosciences, Bengaluru, India; ^2^Department of Medicinal Chemistry, National Institute of Pharmaceutical Education and Research, Hyderabad, India; ^3^Department of Microbiology and Applied Sciences, University of Agricultural Sciences, Bangalore, India

**Keywords:** PET molecular imaging, drug development, radioligands, fluorine-18, carbon-11

## Abstract

Positron emission tomography with selective radioligands advances the drug discovery and development process by revealing information about target engagement, proof of mechanism, pharmacokinetic and pharmacodynamic profiles. Positron emission tomography (PET) is an essential and highly significant tool to study therapeutic drug development, dose regimen, and the drug plasma concentrations of new drug candidates. Selective radioligands bring up target-specific information in several disease states including cancer, cardiovascular, and neurological conditions by quantifying various rates of biological processes with PET, which are associated with its physiological changes in living subjects, thus it reveals disease progression and also advances the clinical investigation. This study explores the major roles, applications, and advances of PET molecular imaging in drug discovery and development process with a wide range of radiochemistry as well as clinical outcomes of positron-emitting carbon-11 and fluorine-18 radiotracers.

## Introduction

**Figure 1:**
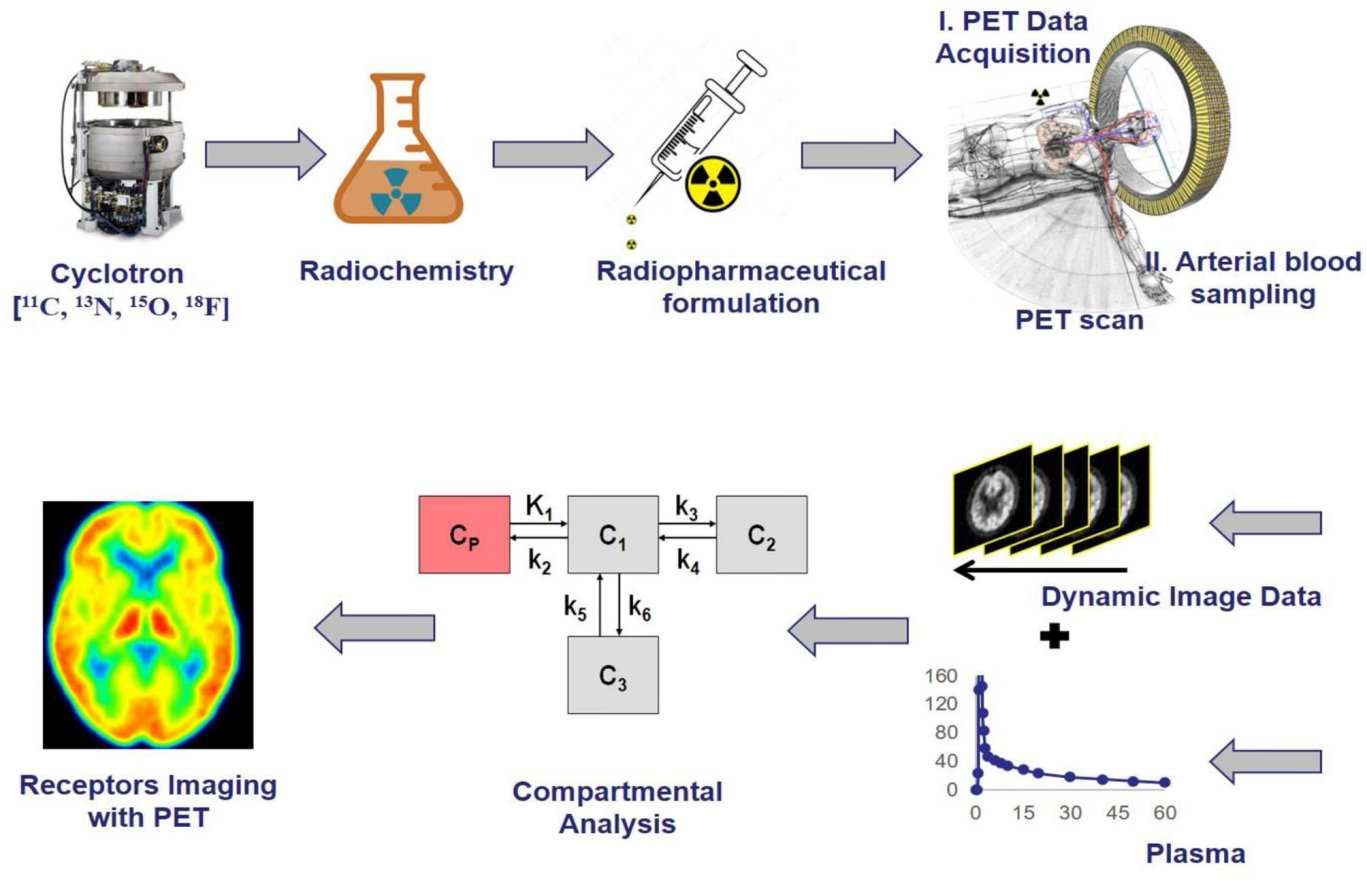
The schematic representation of receptor imaging with positron emission tomography (PET) and the flow from radionuclide generation to PET Imaging output, where the initial step is the generation of positron-emitting radionuclides, the second step is the radiochemical synthesis, the third step is the preparation of suitable radiopharmaceutical formulation, the fourth step is the PET imaging data acquisition, the fifth step is the PET data pre and post-processing using software technologies, the last stage is the output of PET images reveal physiological changes based on radiotracer uptake corresponding to the intensity and dynamics in the color of images.

The selective radioligands used for positron emission tomography molecular imaging have vastly increased over the past decade because of many advances in biomedical research as well as clinical investigation (1). It has been estimated that radiopharmaceuticals with 39.1% and radiodiagnostics with 7% were used in the 5 years from 2020 to 2025 by the *MEDrays Intell* nuclear medicine world market report 2019 (2). Positron emission tomography (PET) molecular imaging with radioligand acts like precision pharmacology, provide accurate information related to therapeutic drug development in living subjects *in vivo* like determining dose and dosing interval, a biomarker for drug efficacy, identifying homogeneous groups whether a new drug reaches the specific region of interest in the body with an effective ED_50_ concentration, specific/non-specific receptor binding, receptor occupancy, quantitative relationship, brain penetration, metabolism in the brain as well as peripheral, which could enhance clinical trials and biomedical research involving animal studies with more reliable data (3). The PET imaging modality visualizes and measures rates of various physiological processes at the molecular level *in vivo* such as glucose metabolism, neurotransmitters, uptakes, and blood flow, etc. in cancer, cardiovascular, and various neurological conditions (4). The basic principle of PET is the detection of two annihilated photons traveling in opposite directions at 180° through the collision of an emitted positron from a radiotracer with a local electron in the living system, followed by giving medical images which map the location of radiotracer in the body (5). The PET is very sensitive because it detects photons from tracer quantity of radioligand in picomolar to nanomolar range, and which do not show any pharmacological responses due to lower concentration than ED_50_ values, and the radiation exposure to a patient is also minimal (6). Its major limitation is to provide low anatomical information, whereas CT, and MRI provide good anatomical data, therefore PET is combined with other molecular imaging modalities and emerged as multimodality devices like PET-CT, and PET-MRI to provide both functional and anatomical data simultaneously. The spatial resolution of PET is 2–6 mm but the MRI has <<1 mm, whereas the sensitivity of PET is 10^−12^ M, but the MRI has only 10^−4^ M, hence it has higher significance in the field of medical imaging (7). To enable good performance *in vivo*, each radioligand should meet the criteria which include lipophilicity (log D) in the range of 1 ≤ log D ≤ 3, molecular weight < 500 daltons, number of hydrogen bond donors is <1, No active P-glycoprotein-mediated efflux pump, Affinity based on dissociation/inhibition constant (K_d_/K_i_) is <10 nM, and the receptor density to affinity ratio should be >10 (8). Several fluorine-18 and carbon-11 radiotracers are being used currently in various biomedical research programs (preclinical studies) as well as in clinical PET centers (clinical studies), in which the fluorine-18 radiotracers include ^18^F-fluorodeoxyglucose for glucose metabolism, ^18^F-fluorothymidine for tumor cell proliferation, ^18^F-fluoromizonidazole for tumor hypoxia, ^18^F-fluoromisonidazole for myocardial ischemia, ^18^F-fluorodopa for Dopamine neuronal densities, ^18^F-flumazenil for benzodiazepine receptors densities, ^18^F-estradiol for estrogen receptor expression, whereas the carbon-11 radiotracers include ^11^C-methionine for amino acid transport and protein synthesis, 2-[^11^C]thymidine for tumor cell proliferation, ^11^C-acetate for the tricarboxylic acid cycle, [^11^C]meta-hydroxyephedrine for presynaptic catecholamine reuptake, ^11^C-raclopride for dopamine receptor densities, ^11^C-methylspiperone for 5-HT2 receptor density, and ^11^C-PK11195 for neuronal microglial activation (9, 10). A generalized view of radiochemistry and PET molecular imaging studies has been shown in Figure 1, where the initial step focuses on generating positron-emitting radionuclides like carbon-11 and fluorine-18 using different targets in a cyclotron, the second step is critical and it involves the radiochemical synthesis by transferring radioactivity into either automated modules like GE Tracerlab MX, FX_2_C, FX_2_N or hot cell apparatus where the mixture of selective precursor for radiolabeling and solvents are present in a heating vial, and this step should adhere with cGMP compliances for human applications, and the third step focuses on the preparation of suitable radiopharmaceutical formulation for injection into a living subject after conformation with quality control studies like pH, endotoxin levels, purity, molar activity, solvent levels, etc. (11). The fourth step is the PET imaging data acquisition by setting up required parameters for different studies like static or dynamic PET studies, where simultaneous arterial blood sampling is required for dynamic PET study to quantify the available receptor concentration in living subjects. The majority of clinical PET centers use static PET studies just to monitor the physiological changes in different diseases conditions like oncology, neurology, and cardiology. The fifth step is the PET data pre- and post-processing for both static and dynamic studies, the parameters include motion correction, co-registration of MRI with PET, spatial normalization, generation of the region of interest (ROI), and partial volume correction (PVC). The static PET data analysis gives standardized uptake values (SUV), which differentiate patient conditions from healthy controls, whereas the dynamic PET data analysis provide pharmacokinetic and pharmacodynamic profiles of radiotracers like the volume of distribution, binding potential, metabolism, etc. using different methods like tissue compartmental analysis, spectral analysis, and mathematical graphical analysis. The last stage is the PET images of living subjects' brains or whole bodies with color-coding to observe the radiotracer uptake in the specific regions of the body, which provides information about the physiological changes in any disease condition compared to normal healthy controls (12).

The United States Food and Drugs Administration (USFDA) does not have regulations to monitor the production of PET drugs for research purposes, since they do not come under the drugs and cosmetic act, which outlines that they should exhibit pharmacological actions at therapeutic doses. The investigational use of PET drugs are subject to Investigation of New Drug (IND) approval to check the efficacy and safety of new PET drugs for clinical use, whereas research use of PET drugs does not need the approval of IND, but Radioactive Drug Research Committee (RDRC) approval is necessary to check basic information about the physiochemical properties, metabolism, pathophysiology, biochemistry or pharmacology of new PET drugs for clinical use. Section 121C/1A of the Modernization Act by the FDA focuses on IND approval processes and current good manufacturing practice (CGMP) requirements for PET drugs to be used clinically. Section 21 CFR 361.1 by FDA regulations describes conditions for PET drugs for research use that are exempted from approval of IND since they are considered safe and effective to use for research studies but not for clinical trials by RDRC with an approval. Some of the PET drugs for clinical use in compliance with CGMP regulations and United States Pharmacopeia (USP) monographs/standards include [^13^N]NH_3_, [^11^C]CO, [^11^C]raclopride, [^11^C]methionine, [^11^C]flumazenil, [^11^C]mespiperone, [^11^C]sodium acetate, [^18^F]FDG, [^18^F]Fluorodopa, [^18^F]sodium fluoride, [^15^O]H_2_O (13, 14).

**Figure 2:**
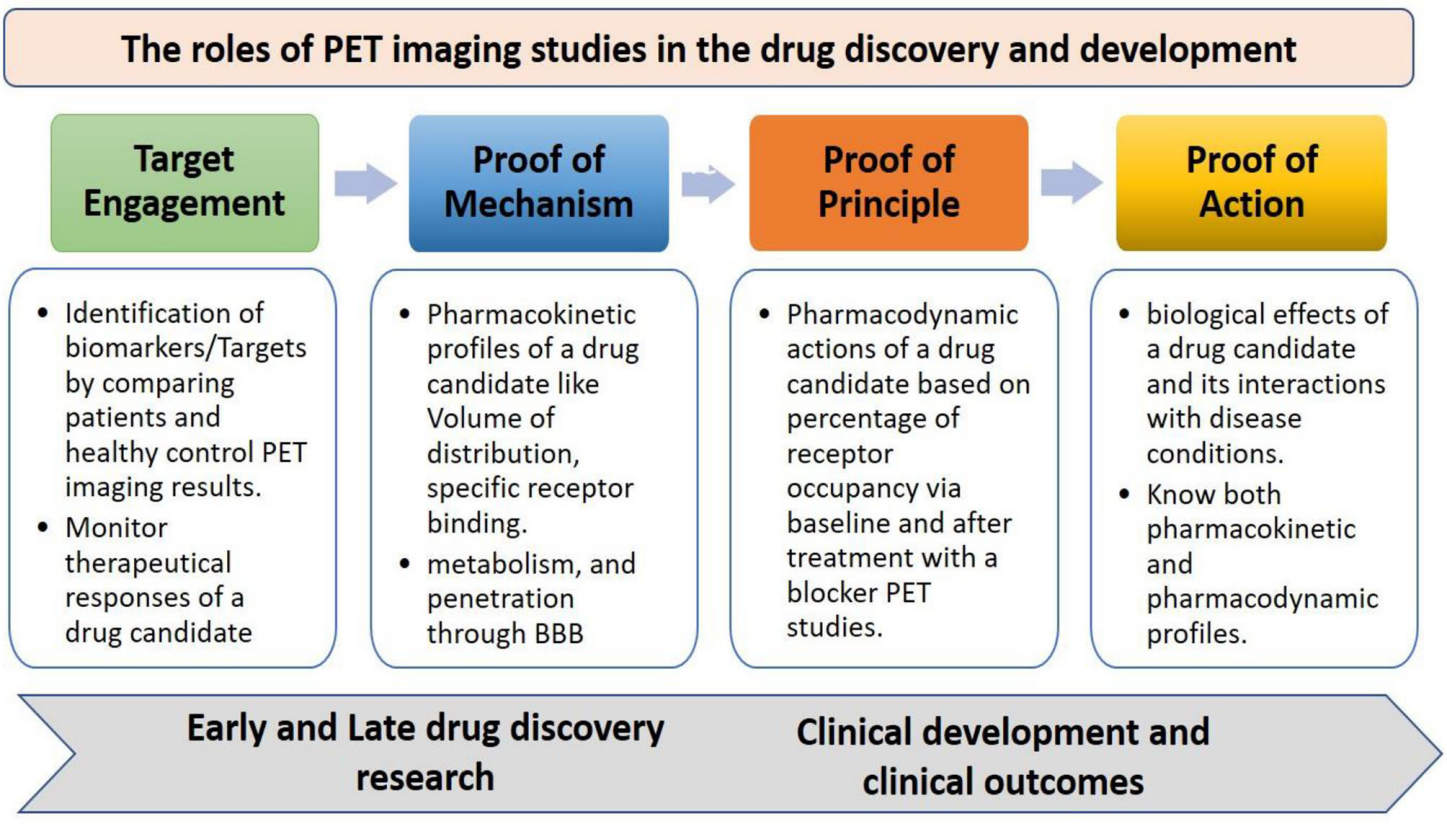
The roles of PET imaging studies in drug discovery and development, the PET advances the development of therapeutic drugs with various approaches like target engagement, proof of mechanism, proof of principle, and proof of concept at different stages of early and late-stage drug discovery research, and clinical development process.

The traditional preclinical and clinical studies without any advancements have shown very low success rate in the drug development process since many factors contribute to the difficulty of developing novel therapeutic drugs, which include the low optimal level of drug-receptor binding at the target, non-specific binding, lack of expected therapeutic efficacy or biological effects of a drug candidate, severe side effects, and drug metabolism. Therefore, there have been several advancements in the drug discovery and development process to overcome the limitations as well as increase the success rate, in which the best and most popular technique commonly used in drug discovery is the PET tool, it can facilitate the development of therapeutic drugs at a faster rate based on various approaches like target engagement, proof of mechanism, proof of principle, and proof of concept (Figure 2) (15, 16). The PET has many roles in the early and late-stage drug discovery and development process like in different stages of preclinical and clinical trials by revealing information about pharmacokinetic profiles as well as pharmacodynamic actions of a new drug candidate in a living system (17).

**Figure 3:**
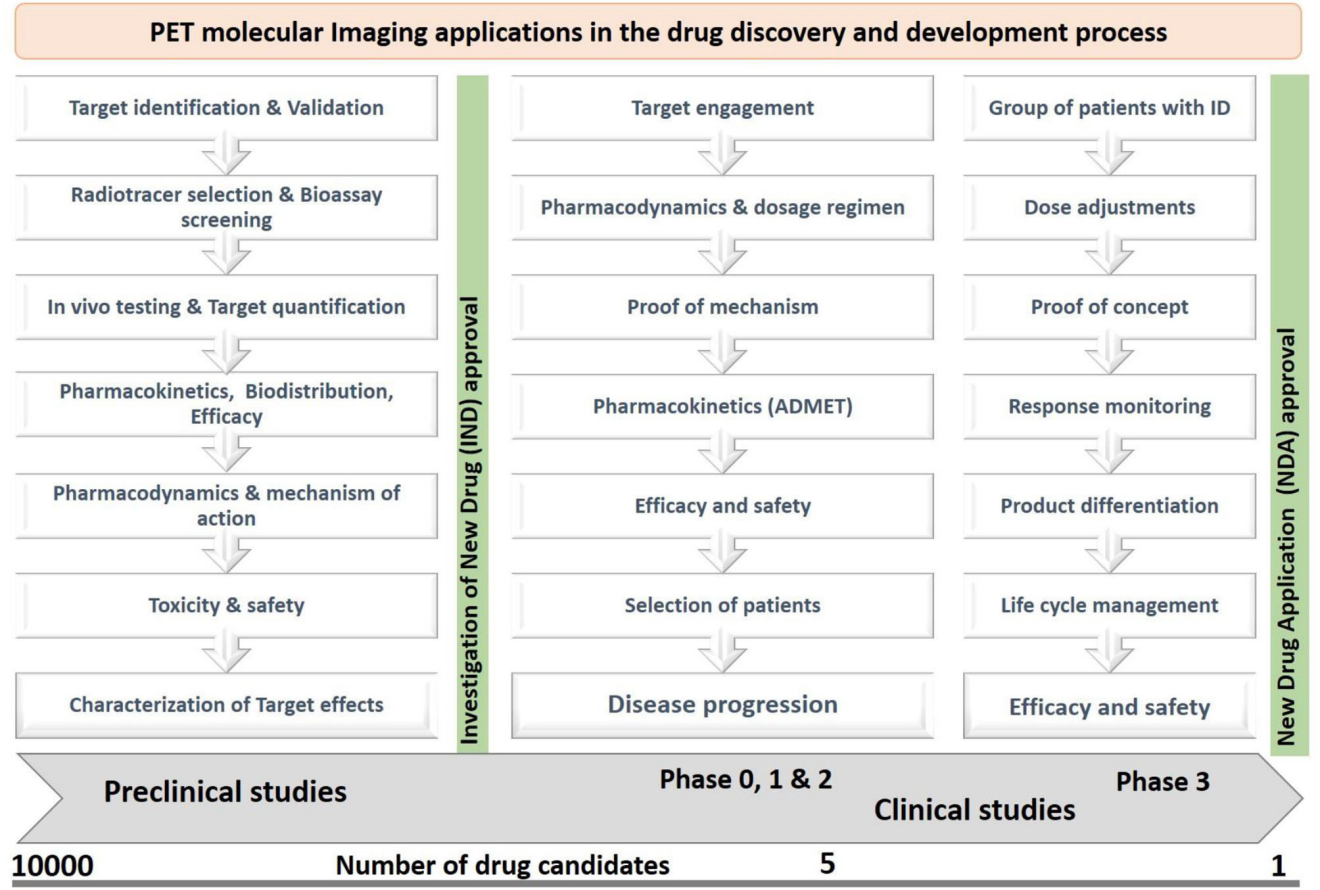
The PET imaging applications in drug discovery and development, where the preclinical and clinical studies focused on pharmacokinetic profiles, pharmacodynamic actions, safety, efficacy, toxicity, dosage regimen, and response monitoring of a new drug candidate by studying behaviors of a radiotracer in living subjects.

The target engagement approach helps in finding key biological targets or biomarkers in various disease conditions, which can be identified by cross-sectional studies or longitudinal studies. The identified biological targets or biomarkers can also be used to monitor the therapeutic responses of a drug candidate. The proof of mechanism approach helps in finding specific binding of a drug candidate with biological targets or biomarkers through its volume of distribution and kinetic rates, metabolism in the brain, and penetration through the blood-brain barrier. The proof-of-concept approach helps in understanding the biological effects of a drug candidate and its interactions with receptors in various diseases conditions (18, 19). PET plays a crucial role in the early and late-stage preclinical studies to increase the drug development process by providing information on the key biological targets or biomarkers, selection of a suitable radiotracer, biodistribution, target quantification, pharmacokinetics, efficacy, safety, and toxicology profiles (20, 21), The PET imaging applications at various stages of the drug discovery and development process are shown in Figure 3.

## Quantitative Receptor Binding and Biodistribution Studies Using PET

Quantitative receptor binding and biodistribution are the major applications of PET molecular imaging in the drug discovery and development process. PET provides a change in the concentration of receptors in various disease conditions based on the quantitative measurement of receptor density, and biodistribution using different compartment models. The *in vitro* radioligand binding assays are the basis to translate its techniques into PET molecular imaging like receptors imaging *in vivo*, which are mainly based on the equilibrium between the bound ligand-receptor complex (LR) and a free receptor (R) as well as a free ligand (F) with reaction rate constants k_on_ and k_off_ follow the law of mass action (22).


(1) $$\begin{array}{l} L+R\underset{koff}{\overset{kon}{⇌}}LR \end{array}$$

L = Ligand

R = Receptor

LR = bound radioligand

Rate of association = *kon* [L] [R], Rate of dissociation = *koff* [LR]

At equilibrium: association = dissociation


$$\begin{array}{l} kon\;\left[L\right]\left[R\right]=koff\;\left[LR\right] \end{array}$$

the equilibrium dissociation constant (**K**_**D**_) is derived from rate constants *kon*, and *koff*.


(2) $$\begin{array}{l} \frac{koff}{kon}=\left[L\right]\left[R\right]/\left[LR\right]=K_{D} \end{array}$$

Based on *in vitro* radioligand binding as well as dissociation constant (K_D_) for the first time, the term binding potential was introduced into *in vivo* PET molecular imaging. It is based on two parameters such as receptor density, and radioligand affinity toward the target to measure quantitatively the radioligand uptake in the brain. Therefore, it is considered that the binding potential (BP) is the ratio of receptor density (B_max_) to radioligand equilibrium dissociation constant (K_D_). Since the radioligand affinity is the inverse of the equilibrium dissociation constant (K_D_), the BP is also considered as a product of B_max_ and affinity (23). The *in vivo* binding potential quantifies a receptor population with the specific binding of radioligand to the receptor by omitting the free state of radioligand and non-specific binding, and the tracer quantity of radioligand occupies only <5–10% of the target but quantifies the entire receptor population through specific radioligand binding (24).


(3) $$\begin{array}{l} {\text{B}}_{\text{max}}=[\text{LR}]+[\text{R}] \end{array}$$

F = free radioligand = [L]

B = bound radioligand = [LR]


(4) $$\begin{array}{l} Equilibrium\;Michaelis-Menten\;:\;s\;B=B{\text{ max}}^{*}F/K_{D}+F \end{array}$$

(5) $$\begin{array}{l} \text{Rearrange }:\;B/F=B\text{max}/K_{D}+F \end{array}$$

In a PET study, a tracer quantity of radioligand is injected (KD >> F), and the Equation (5) is rearranged as below,


(6) $$\begin{array}{l} B/F=B_{\max}/K_{D}=B_{\max}*affinity \end{array}$$

Therefore, Radiotracer Binding Potential = B/ F = B_max_ / K_D_.

The term B_max_ has been commonly used in radioligand binding assays *in vitro* due to the homogeneity of all available receptors, where the *in vivo* conditions show heterogenicity of receptors due to competition between radioligand and endogenous neurotransmitters for receptor binding. Hence, the term B_max_ has been replaced with B_avail_ (total available receptor concentration) for *in vivo* PET imaging studies. The *in vivo* binding potential (BP) quantifies the ratio of specific radioligand binding to other reference concentrations like free radioligand, bound with plasma proteins or non-displaceable binding at equilibrium, therefore, it was categorized into three individual binding potentials based on different reference concentrations of radioligand like BP_F_, BP_P_, and BP_ND._ The BP_F_ refers to the ratio of the concentration of specific radioligand binding (Cs) to the concentration of free radioligand (C_F_) at equilibrium. The BP_P_ refers to the ratio of the concentration of specific radioligand binding (C_S_) to the concentration of both free and plasma protein-bound radioligand (C_P_) at equilibrium. The BP_ND_ refers to the ratio of the concentration of specific radioligand binding (Cs) to the concentration of non-displaceable uptake (C_ND_). The below mathematical equations help us to understand different binding potential values with respect to reference concentrations of radioligand (25, 26). The BP_F_ and BP_P_ require measurement of the arterial input function to quantify receptor occupancy, whereas the BP_ND_ does not need blood sampling during PET analysis (27). All the parameters relating to BP_F_, BP_P_, and BP_ND_ are summarized in Table 1.

**Table 1:** Three different binding potential values and their associated factors.

**Parameter**	**Ratio**	**Reference conc**.	**Rate constants**	**PET**	**Plasma parent**	**Free fraction (*f*_P_)**	**Result**
*BP*_ND_	C_s_/C_ND_	Non-displaceable uptake	$\underset{k_{4}}{\overset{k_{3}}{⇌}}$	√	x	x	$\frac{B_{avail}}{K_{D}}*f_{ND}$
*BP*_P_	C_S_/C_P_	Total plasma	* $\underset{k_{2}k_{4}}{\overset{k_{1}k_{3}}{⇌}}$ *	√	√	x	$\frac{Bavail}{K_{D}}*f_{P}$
*BP*_F_	C_S_/C_fp_	Free plasma	* $\underset{fpk_{2}k_{4}}{\overset{k_{1}k_{3}}{⇌}}$ *	√	√	√	$\frac{B_{avail}}{K_{D}}$

*B_avail_, Density of receptors available to bind radioligand but some receptors may be unavailable; bound by the transmitter or sequestered; f_P_, free fraction in plasma; f_ND_, free fraction in the nondisplaceable compartment*.

## Volumes of Distribution

Determining the volume of distribution (*V*_T_ in mL.cm^−3^) is an essential step in the drug discovery and development process to establish the different binding potential values corresponding to receptor densities based on the Area Under Curves (AUC) as well as kinetic rate constants. *V*_T_ is the ratio at the equilibrium of the concentration of radioligand in the brain and the plasma. In general, *V*_T_ refers to an uptake of radioligand in the brain relative to how much radioligand is delivered *via* arterial plasma. *V*_T_ is calculated by the area brain curve divided by the area plasma curve at the equilibrium. *V*_T_ is always independent of injected activity (28, 29). The total concentration of radioligand in the tissue (C_T_) is based on the concentration of specifically bound with receptors (S), bound with plasma proteins or non-displaceable binding (ND), and free state in the tissue water (F). The volume of distribution measures the ratio of the concentration of radioligand in each component like specific bound, non-specific bound, or free radioligand in tissue water at equilibrium to the concentration of parent radioligand (C_P_) in plasma, which is separated from radiometabolites by HPLC analysis. The *V*_*S*_ refers to the ratio of the concentration of specific radioligand binding (Cs) to the concentration of parent radioligand (C_P_) in plasma free from radiometabolites at equilibrium. The *V*_*ND*_ refers to the ratio of the concentration of non-displaceable uptake (C_ND_) to the concentration of parent radioligand (C_P_) in plasma free from radiometabolites at equilibrium. The *V*_*T*_ refers to the ratio of the concentration of radioligand in tissue (C_T_) to the concentration of parent radioligand (C_P_) in plasma free from radiometabolites at equilibrium. The below mathematical equations help us to understand different *V*_*T*_ values with respect to reference concentrations of radioligand (30, 31). All the parameters related to V_S_, V_ND_, and V_T_ are summarized in Table 2.


(7) $$\begin{array}{l} \text{Here},C_{\text{T}}=C_{\text{S}}+C_{\text{ND}}+C_{\text{FT}} \end{array}$$

The *V*_S_ and BP_P_ refer same as the ratio of the concentration of specific radioligand binding (C_S_) to the concentration of both free and plasma protein-bound radioligand (C_P_) without radiometabolites at equilibrium (32). The *V*_*T*_ and *V*_*ND*_ are calculated from specific region-of-interest using different reference tissue methods as an alternative to the arterial input function. In general, the ratio of V_T_/V_ND_ is referred to as the distribution volume ratio (DVR), it is commonly used as a function of receptor availability and it requires arterial input function, but many methods have been developed to calculate DVR without the need for blood sampling with different reference tissue methods (33). The individual binding potential values are calculated using the volume of distribution, which in turn depends on rate constate values as shown in Table 3.

**Table 2:** Three different parameters of distribution volume and their associated factors.

**Parameter**	**Ratio**	**Reference conc**.	**Rate constants**	**PET**	**Plasma parent**	**Free fraction (*f*_P_)**
**V**_ND_	C_ND_/C_P_	Non-displaceable uptake	$\underset{k_{2}}{\overset{k_{1}}{⇌}}$	√	√	x
**Vs**	C_S_/C_P_	Specific bound	* $\underset{k_{2}k_{4}}{\overset{k_{1}k_{3}}{⇌}}$ *	√	√	X
**V**_T_	C_T_/C_P_	Total tissue	*$\underset{k_{2}}{\overset{k_{1}}{⇌}}$*(1+*$\underset{k_{4}}{\overset{k_{3}}{⇌}}$*)	√	√	x

**Table 3:** The relationship of rate constants to the volume of distribution and binding potential.

**Binding Potential**	**← Volume of distribution**	**← Rate constants**
*BP*_ND_	(V_T_-V_ND_)/V_ND_	$\underset{k_{4}}{\overset{k_{3}}{⇌}}$
*BP*_P_	(V_T_-V_ND_)	$\underset{k_{2}k_{4}}{\overset{k_{1}k_{3}}{⇌}}$
*BP*_F_	(V_T_-V_ND_)/*f*_*P*_	$\underset{fpk_{2}k_{4}}{\overset{k_{1}k_{3}}{⇌}}$

**Figure 4:**
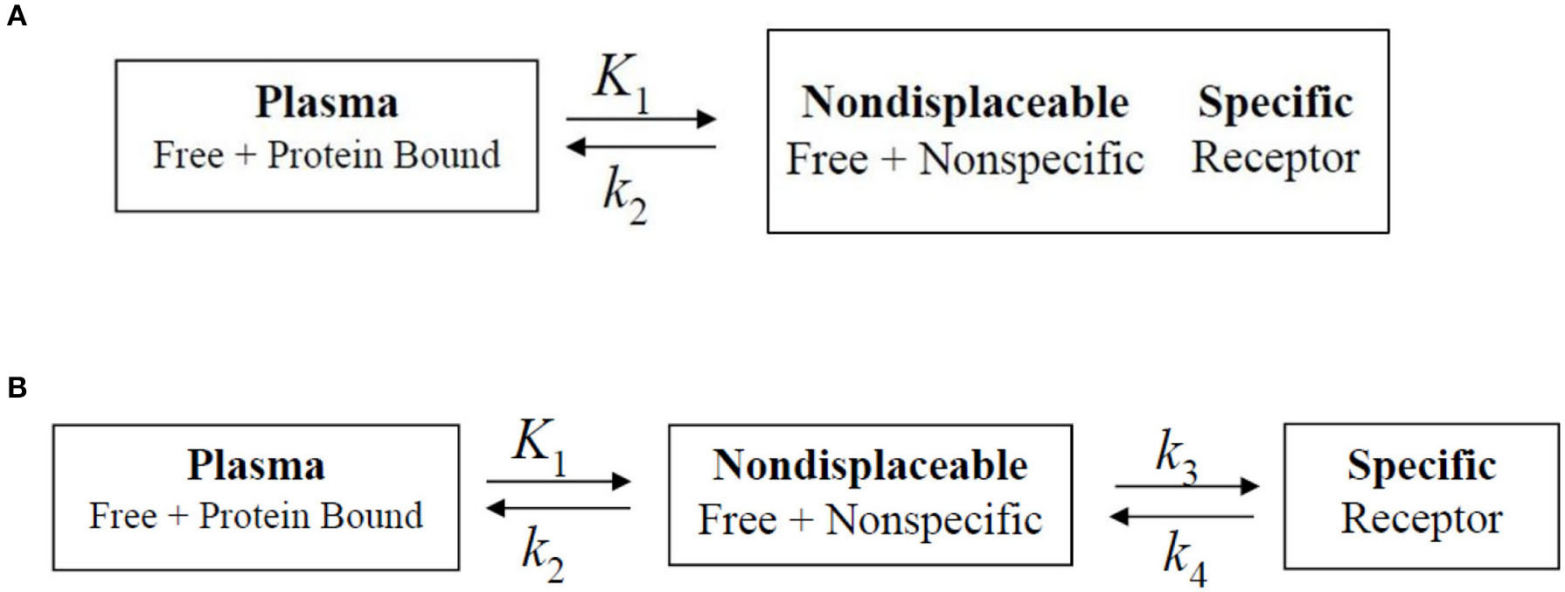
Biopharmaceutical compartment analysis models. **(A)** One-tissue compartment, in which the whole tissue is considered as one compartment including the non-displaceable (free plus non-specific) along with the specifically bound. **(B)** Two-tissue compartment. In which two compartments are present in the tissue which include non-displaceable (free plus non-specific) and specific bound. The rate constants are generally obtained *via* dissociation constant from the tissue to the plasma.

**Figure 5:**
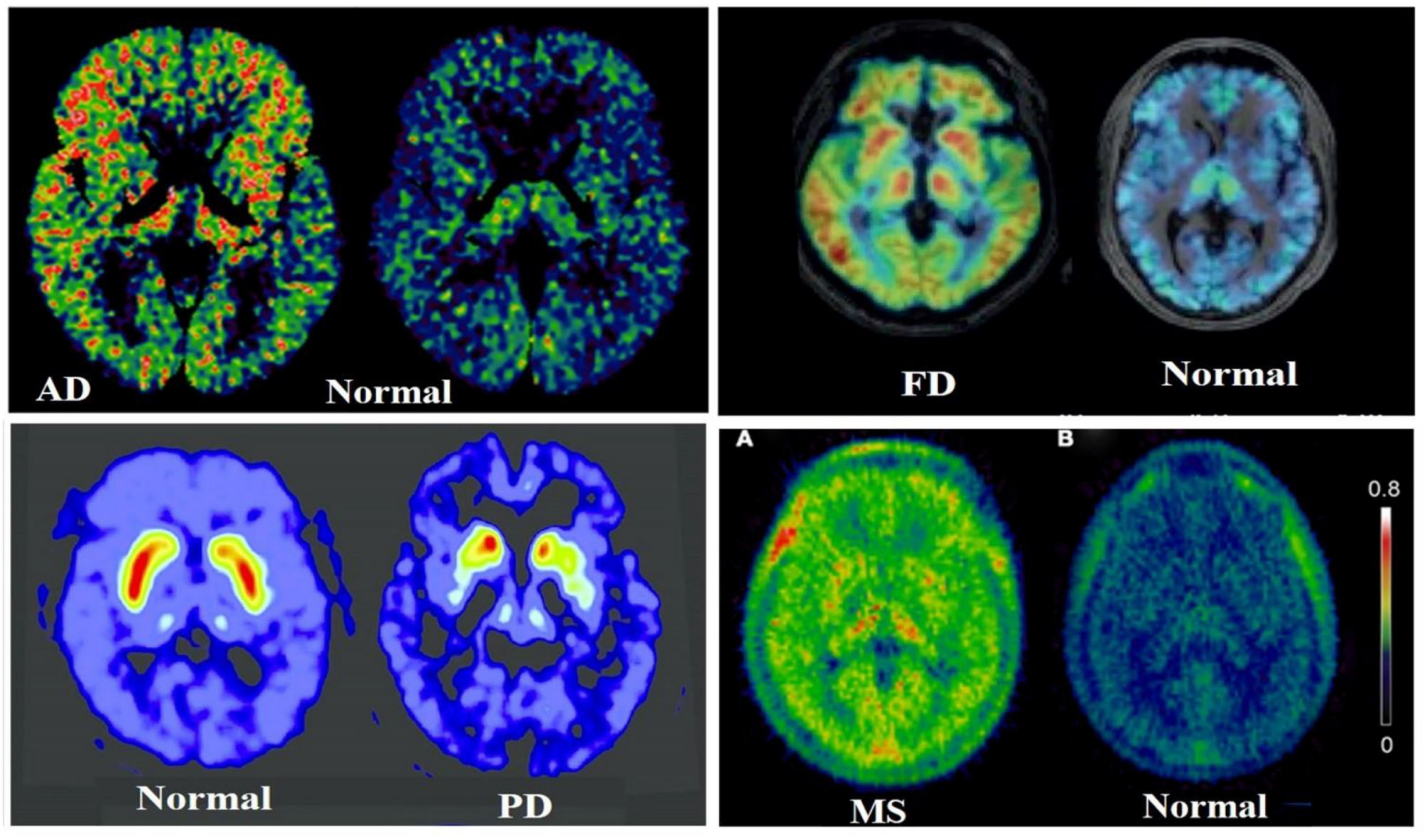
Illustrates a clear differentiation between the abnormal conditions in the brain from a healthy human brain control using PET molecular imaging tools with selective radioligand, herein a few best examples were taken from (36–39), such as [^11^C]PBR28 showed high binding in different cortical regions of Alzheimer's Disease (AD) patient compared with a normal brain, the [^11^C]PBR28 showed increased binding in frontal and temporal lobes in Frontotemporal Dementia (FD) compared with healthy controls, the [^18^F]FDOPA showed a little uptake in regions of caudate and putamen corresponding to nigrostriatal pathway in Parkinson's Disease (PD) patient from the healthy brain, and the [^11^C]PK11195 showed uptake in the regions with high activation of microglia in a Multiple Sclerosis (MS) patient compared to a healthy normal brain.

The rate constant values are based on two-tissue compartment models (Figure 4), here these rate constant values provide the volume of distribution, which in turn provide binding potential values based on receptor density and dissociation constant. In the one-tissue compartment (1TCM) the whole tissue is considered as one compartment including the non-displaceable (free plus non-specific) along with the specifically bound, whereas in the two-tissue compartment (2TCM), two compartments are present in the tissue which include non-displaceable (free plus non-specific) and specifically bound (34, 35). The 2TCM is a commonly used model to obtain the volume of distribution and binding potential. Figure 5 illustrates a variety of examples on PET imaging of the human brain with abnormal conditions as well as normal healthy brain using different radioligands such [^11^C]-PBR28 showed high binding in different cortical regions of Alzheimer's disease patient compared with a normal brain, [18F]-FDOPA showed a little uptake in regions of caudate and putamen corresponding to nigrostriatal pathway in Parkinson's disease patient from the healthy brain, (11) C-PK11195 showed uptake in the regions with high actation of microglia in a multiple sclerosis patients compared to a healthy normal brain, and the (11) C-PBR28 showed increased binding in frontal and temporal lobes in frontotemporal dementia compared with healthy controls (36–39).

## Receptor Occupancy Studies Using PET

**Figure 6:**
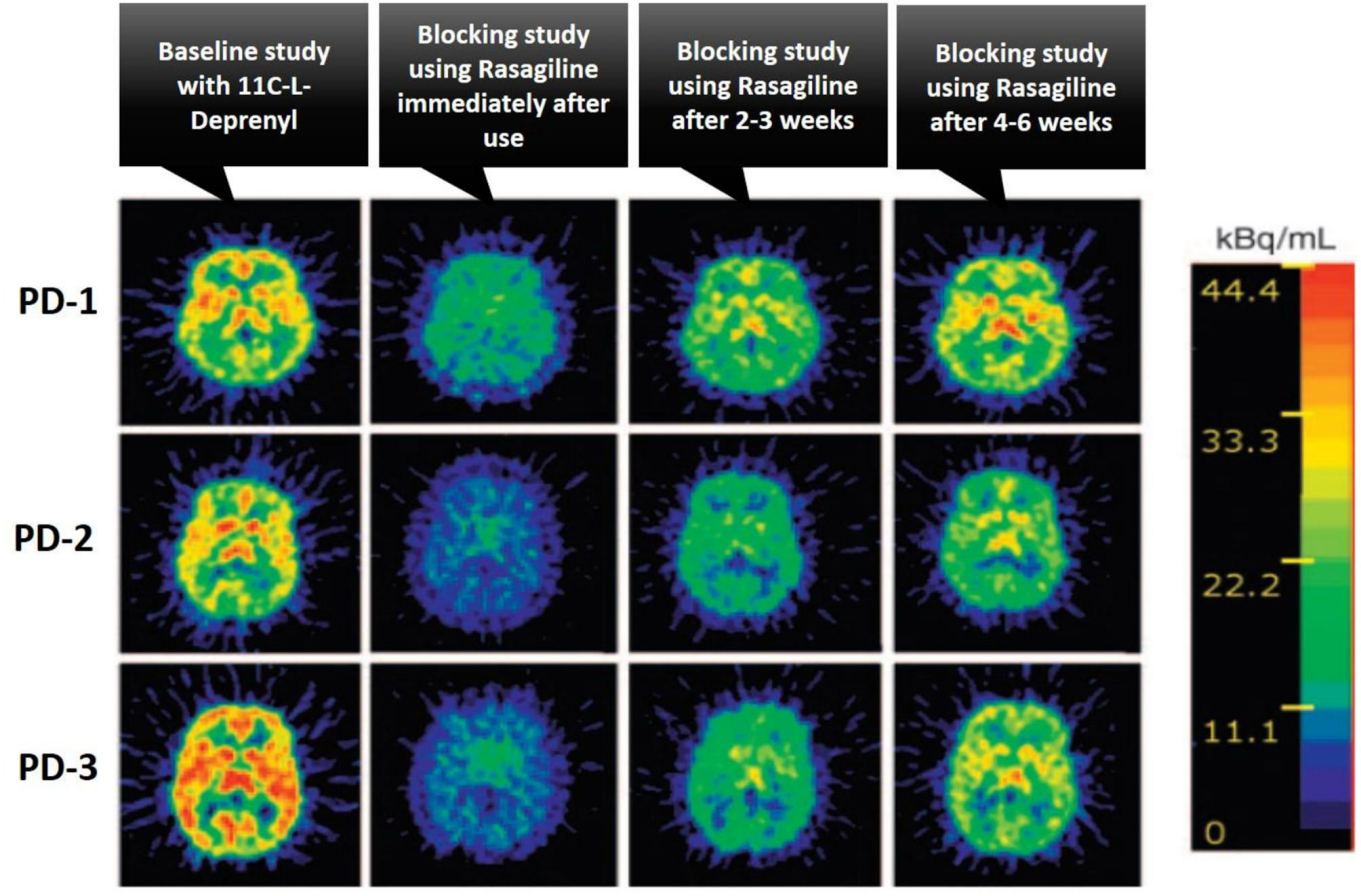
The illustration of the treatment response of Rasagiline drug as a selective MAO-B inhibitor in patients with Parkinson's Disease (PD) using PET imaging studies, where the baseline and after treatment with a blocking agent PET studies were performed to calculate the percentage of receptor occupancy of the brain Monoamine Oxidase B (MAO-B) receptor by rasagiline using the selective radiotracer ^11^C-L-Deprenyl, three different PD patients were taken for the studies and performed initial baseline PET study with ^11^C-L-Deprenyl, followed by PET study with blocking agent rasagiline at different time intervals like immediate use, after 2–3 and 4–6 weeks to monitor the treatment response in PD condition, this case study was taken from (42).

The drug-receptor occupancy is one of the major applications of PET molecular imaging in the drug discovery and development process. Since PET is ready to provide receptor densities with selective radioligand, it can also easily test the efficacy of new drug candidates by measuring the plasma concentration with the receptor occupancy in a time course (40). This data provides a lot of pharmacological information of a new drug in the development process like the penetration capability across the blood-brain barrier, specific binding with the target, and rational dosage selection in different phases of drug development. The drug-receptor occupancy gives us a piece of information about the treatment-induced relative change in the concentration of available receptors, it can be easily calculated with the relative change in the binding potential before and after the drug treatment using PET baseline and blocking studies (41). The drug-receptor occupancy (RO%) is calculated using the below formula, and Figure 6 illustrates PET images of the human brain in Parkinson's Disease (PD) condition with baseline and after treatment with a blocking agent study, these studies were performed to measure the brain Monoamine Oxidase B (MAO-B) receptor occupancy by Rasagiline (selective MAO-B inhibitor) using the selective radiotracer ^11^C-L-Deprenyl, and also showed treatment response in PD condition with selective drug Rasagiline (42).


(8) $$\begin{array}{l} RO\left(\%\right)=1-\frac{{\text{B}}_{\text{Avail}}\left(\text{After Treatment}\right)}{{\text{B}}_{\text{Avail}}\left(\text{Baseline}\right)} \end{array}$$

## The Chemistry of Radiolabeling With Fluorine-18 and Carbon-11 for PET Imaging

### Radiosynthesis Using Fluorine-18

The fluorine-18 is a commonly used radionuclide because of its maximum half-life of 110 min, high radiochemical yields, easy access to PET clinical centers, and best PET imaging properties (43). The [^18^F]FDG is regarded as a potential PET radiotracer for imaging and quantifying various physiological changes in cancer, cardiovascular and neurological conditions. The number of research papers on the development of fluorine-18 based PET radiotracers have been increasing exponentially, and many cGMP compliance cyclotron facility centers were set up to produce ^18^F-radiopharmaceuticals and distribution to nearby PET clinical centers (44). Many clinical and research centers produce fluorine-18 radionuclide with high molar activity through cyclotron facility using ^18^O water, which is irradiated in a metal target like titanium, niobium, silver, etc. with a proton through a source under high energy in the range of 3–20 MeV *via* nuclear reaction [^18^O (p, n) ^18^F]. In this nuclear reaction, one proton combines with ^18^O water at high energy during irradiation to produce ^18^F fluoride by eliminating a neutron to balance this nuclear reaction (45). The produced ^18^F fluoride comes as ^18^F in ^18^O water and is used for ^18^F-radiopharmaceuticals development. The positrons emitted by a radioligand interact with local electrons in the body and cause annihilation reactions, through which two photons (gamma radiation) are generated with the energy of 511 KeV each and travel in the opposite direction at 180° toward PET detectors around a living subject (46). Some cyclotron facilities can also generate fluorine-18 as [^18^F]F_2_ fluorine gas using enriched ^18^O in an F_2_ gas target with high energy protons *via* nuclear reaction [^18^O (p, n) ^18^F] (47). 18F-radiotracers travel distance is <0.3 mm path linear range in biological tissues which is lower than other positron emitters. The fluorine-18 with a 110 min half-life can resist long-run radiosynthesis, quality control studies, and whole-body human scans by PET at later hours. The majority of ^18^F-radiopharmaceutical synthesis is automated using FX2C, FX2N, and MX modules to reduce radiation exposure to radiochemists, and scientists (48). The ^18^F fluoride acts as a nucleophile, hence it is used in nucleophilic substitution reactions, whereas [^18^F]F_2_ fluorine acts as an electrophile, hence it is used in electrophilic substitution reactions (49). Recent advances have come to produce ^18^F-radiopharmaceuticals through transitional metal-mediated reactions and prosthetic groups involved reactions for 18F-labeled peptides, and proteins (50).

**Figure 7:**
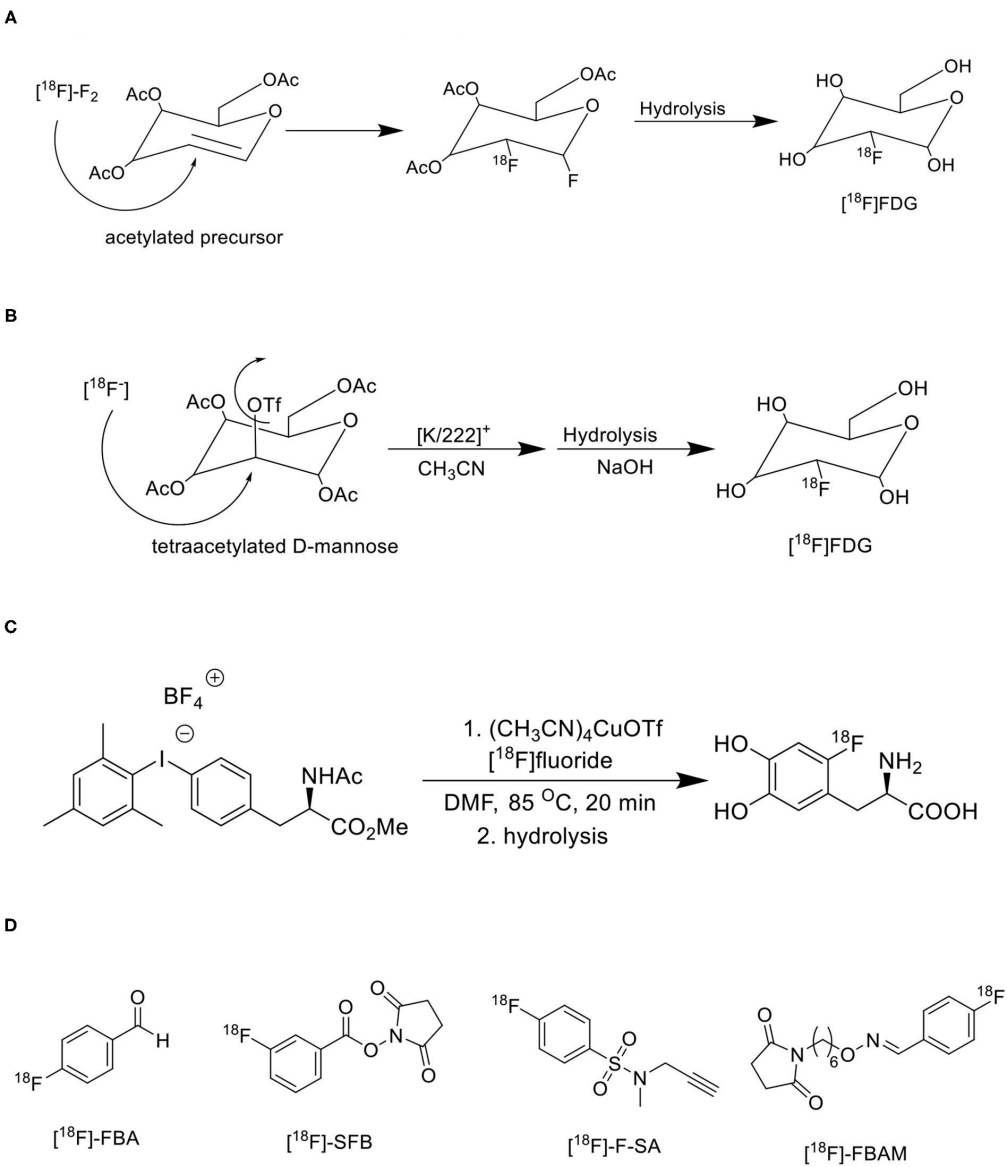
Various synthetic approaches for 18F-radiofluorination of biomolecules. **(A)** Electrophilic radiofluorination of [^18^F]FDG. **(B)** Nucleophilic radiofluorination of [^18^F]FDG. **(C)** Transitional metal-mediated radiofluorination of [^18^F]F-DOPA. **(D)** Commonly used prosthetic groups for radiofluorination.

The electrophilic radiofluorination reactions with [^18^F]F_2_ fluorine are limited since the radiochemical yield is almost 50% due to dilution of 19F fluorine, leakage or contamination is very common as [^18^F]F_2_ fluorine comes as gas from the cyclotron, achieve minimum molar activity (0.05–0.5 GBq/μmol), and [^18^F]F_2_ fluorine is a non-selective electrophilic agent for regiospecific reactions (51). The generation of electrophilic [^18^F]F_2_ fluorine involves two steps, initially, fluorine-18 is generated in a metal target using irradiating ^18^O water with high energy protons (10 MeV), later this fluorine-18 undergoes isotopic exchange with continuous irradiation of a mixture of molecular fluorine and noble-gas (F: Ne = 2: 98) as a gas target, and finally the gaseous form of [^18^F]F_2_ fluorine releases for electrophilic radiofluorination reactions (52). Initially, the clinically used PET radiotracers like [^18^F]FDG, ^18^F-L-DOPA, ^18^F-EF5, and ^18^F-labeled 5-fluorouracil were synthesized using electrophilic radiofluorination reactions with ^18^F-F_2_ isotope gas, from which the synthesis of [^18^F]FDG was shown in Figure 7A (53). Many synthetic challenges limited the use of electrophilic radiofluorination, and automated synthesis modules (54). The nucleophilic radiofluorination reactions with ^18^F fluoride are most common since the radiochemical yield is >90% due to direct insertion of fluorine-18 into precursor, leakage or contamination is very limited as ^18^F fluoride comes as a liquid from the cyclotron, achieve maximum molar activity (500–5,500 GBq/μmol), and ^18^F fluoride is a selective nucleophilic agent for regiospecific reactions (55). The major limitation of ^18^F fluoride is ready to form hydrogen bonds with the ^18^O water and reduces the nucleophilic substitution reaction rate due to its nucleophilicity, but it can be achieved by removal of water by evaporation and use of polar aprotic solvents in radiochemical reactions (56). The phase transfer catalyst (PTC) like the cryptand Kryptofix 2.2.2 in complex with potassium carbonate or tetrabutylammonium cation could enhance the solubility, and nucleophilicity of ^18^F fluoride in radiochemical synthesis (57). The azeotropic distillation method is widely used in radiochemistry to dry the fluoride-cryptand complex with acetonitrile or using a microwave heating method (58). The commonly used polar aprotic solvents include acetonitrile, N, N-dimethylformamide, dimethyl sulfoxide, and dimethylacetamide. Based on the nature of molecules the nucleophilic radiofluorination is possible either for aliphatic or aromatic substitution reactions. The clinically used PET radiotracers like [^18^F]FDG, ^18^F-FLT, ^18^F-FES, and ^18^F-choline were synthesized using nucleophilic radiofluorination reactions with ^18^F-fluoride, from which the synthesis of [^18^F]FDG was shown in Figure 7B (59). Many automated synthesis modules and methodologies are developed based on nucleophilic radiofluorination (60, 61). Recently one study reported first transition metal assisted ^18^F-labeling of heteroaromatic phenols through deoxyfluorination process *via* Ruthenium π-complexes. It is well-established that this transition metal π-complexes lower the electron density of the heteroaromatic phenols and activates these for nucleophilic aromatic substitution. They have translated this method into a fully automated synthesis of tyrosine and β-CFT derivatives in GE tracer lab FX_2_N modules and achieved high radiochemical yields (62). The same research group extended their research into 18F radiolabeling of biomolecules using the same approach of a chemoselective radio-deoxyfluorination of an aromatic amino acid residue, mainly a tyrosine residue for conjugation of biomolecules (63). Various aromatic and heteroaromatic iodonium salt precursors were developed for ^18^F-labeling *via* Cu-mediated radiofluorination, and the utility of iodonium salts has been increasing vastly for the development of various ^18^F-radiopharmaceuticals due to its high radiochemical yield, regioselectivity, high tolerance, and straightforward synthesis (64). Dr. Pike's group has done extensive research on different iodonium salt precursors and developed several useful PET radiotracers for oncology, and neurology using iodonium salts (65, 66). One research group reported Cu-mediated 18F-radiofluorination of diaryliodonium salts using [^18^F]KF and translated this method into clinically used radiotracers like 4-[^18^F]fluorophenylalanine and 6-[^18^F]F-DOPA, and achieved high radiochemical yields as shown in Figure 7C (67). However, nucleophilic radiofluorination has some limitations, mainly for ^18^F-radiolabeling of protein conjugates since peptides, protein conjugates are unstable at higher temperature is being used in nucleophilic radiofluorination (68). Therefore, prosthetic groups have been used to introduce ^18^F-radionuclide into peptides, protein conjugates (Figure 7D). The ^18^F-radiotracers are developed by conjugating ^18^F-labeled precursors containing prosthetic groups with peptides, and proteins under normal conditions, but the limitations include lower yields and multistep process (69).

### Radiosynthesis Using Carbon-11

Like fluorine-18, carbon-11 is also a widely used radionuclide for PET radiotracer development, and it is a short-lived positron emitter with a half-life of 20.4 min and β^+^ emission of 99.8%. The carbon-11 radionuclide can be generated in two different sources from a cyclotron including [^11^C]CO_2_ or [^11^C]CH_4_ using different targets *via* the ^14^N (p, α)^11^C nuclear reaction (70). These generated different forms of carbon-11 radionuclide are inserted into various organic biomolecules during radiochemical synthesis to develop target-based PET radiotracers. The [^11^C]CO_2_ is generated from a target having nitrogen 100 % and oxygen ~0.1–1% with higher activities ~118 GBq and 11–17 MeV energy, the [^11^C]CH_4_ is generated from a target having nitrogen 100% and hydrogen ~ 10% with higher activities ~67 GBq and 18 MeV energy (71). Several convenient methods were adopted to generate [^11^C]CO_2_ or [^11^C]CH_4_ in high molar activity using different targets (72). In general, the No-Carrier-Added (NCA) method was utilized to generate carbon-11 since the specific (GBq/mg)/molar activity (GBq/μmol) is achieved, whereas the fluorine-18 generation mainly the ^18^F-Fluorine (^18^F-F) is generated *via* Carrier-Added (CA) method, hence the specific (GBq/mg)/molar activity (GBq/μmol) is very low due to dilution with non-radioactive fluorine-19 atoms. Therefore, the molar activities (GBq/μmol) of carbon-11 are always higher than ^18^F-Fluorine (^18^F-F) radionuclide (73). The presence of non-radioactive atoms causes saturation of biological targets thereby lowering target-based PET signal and resolution of PET images, hence the NCA method is essential to increase the ratio of radioactive atoms to non-radioactive atoms (74). The molar activities depend on characteristic decay, therefore, is it essential to focus on the radiotracer injection time during a PET study, duration of radiosynthesis, radionuclide generation, and bombardment time (75). In general, a human PET study requires about 500–600 MBq of carbon-11 at the time of injection, whereas for an animal PET studies <200 MBq is sufficient based on body weight (76). The last stage radiochemical synthesis, rapid quality control studies, and fast dispensing for administration to a living subject are essential with carbon-11 since the half-life is about 20 min various carbon-11 radiotracers can be synthesized with different routes by generating primary and secondary precursors. The major advantage of carbon-11 is the direct insertion of carbon-11 synthons into a precursor to synthesize 11C-radiotracers without optimization trials (77).

The primary precursors [^11^C]CO_2_ or [^11^C]CH_4_ are directly synthesized from a cyclotron using specific targets with nitrogen gas, and a trace amount of hydrogen or oxygen *via* the ^14^N(p,α)^11^C nuclear reaction. The radiochemical yields are very good due to the high concentration of nitrogen with low oxygen/hydrogen, whereas other primary precursors such as [^11^C]CN or [^11^C]CO can also be produced by changing target composition, but yields are moderate (78). Many studies reported the direct insertion of [^11^C]CO_2_ into the molecules *via* [^11^C]carboxylation reactions with different organic compounds like organometallic reagents, amines, etc (79). For example, the direct insertion of [^11^C]CO_2_ can generate various carbon-11 functional groups like ^11^C-carboxylic acid, ^11^C-urea, ^11^C-amide, ^11^C-carbamate, and ^11^C-oxazolidinone, which can further be used for developing target-specific 11C -radiotracers like [^11^C]AR-AO14418, is a selective GSK-3β inhibitor where it has ^11^C-urea functional group, [^11^C]Bexarotene, is a retinoid X receptor (RXR) agonist where it has an ^11^C-carboxylic acid functional group, and ([^11^C]GR103545, is a kappa opioid agonist where it has ^11^C-carbamate functional group (80).

**Figure 8:**
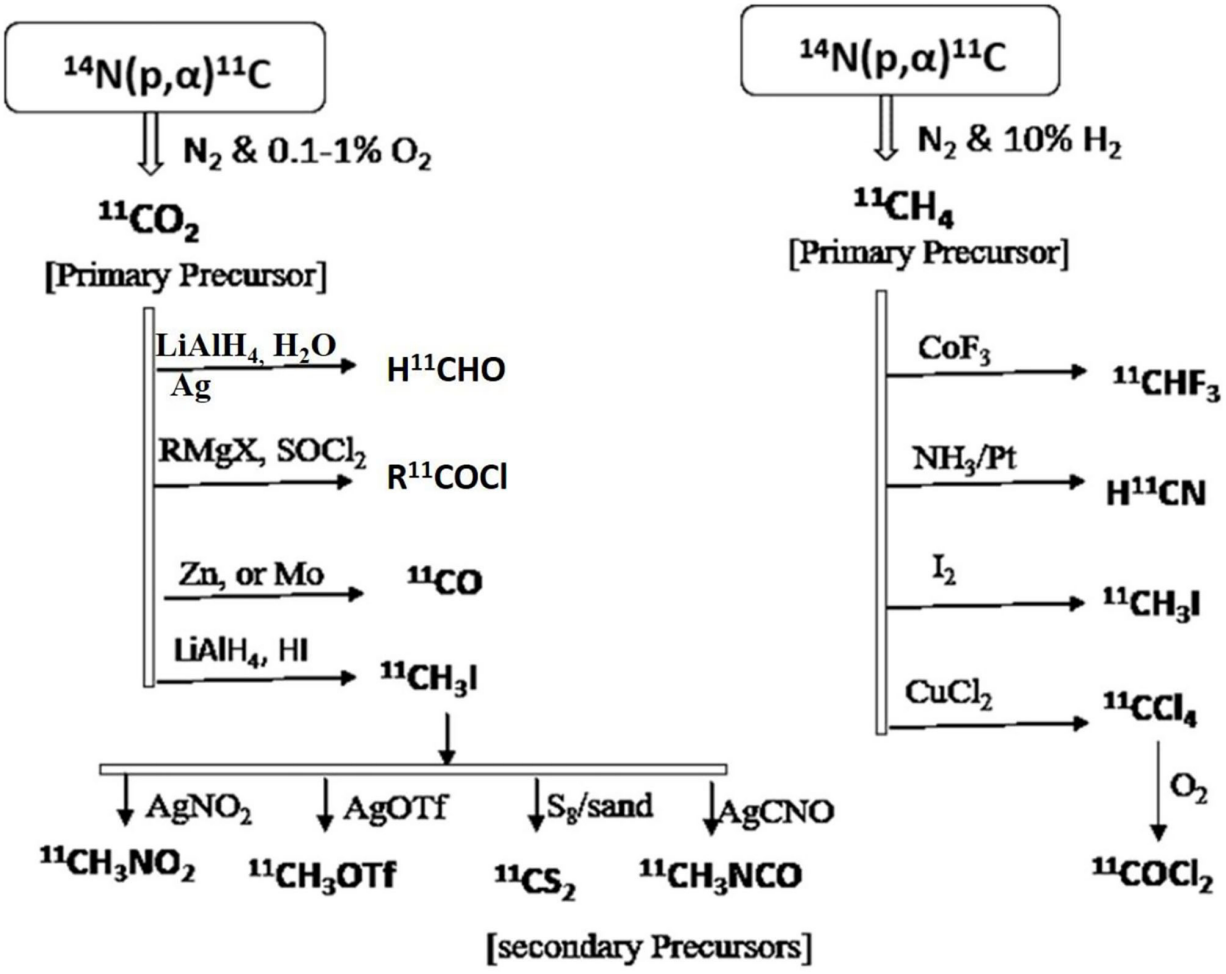
Various synthetic approaches for the generation of secondary precursors to developing carbon-11 inserted biomolecules.

Due to limited access to developing various radioligands with only the primary carbon-11 precursors [^11^C]CO_2_ or [^11^C]CH_4_, many secondary precursors have been developed and used as building blockers to synthesize target-specific radioligand with high accessibility. Many successful radiotracers are used in clinical and preclinical studies are developed using various secondary precursors like ^11^CH_3_I, ^11^CO, ^11^COCl, ^11^COCl_2_, ^11^HCN, ^11^CH_2_O, ^11^CS_2_, and ^11^CH_3_OTf (81). A few reported examples include [^11^C]Raclopride is a cerebral D2 receptors antagonist for imaging Parkinson's diseases, which is synthesized by ^11^CH_3_I precursor, ^11^C(carbonyl)-estramustine phosphate is an estrogen receptors agonist for imaging solid tumors, which is synthesized by ^11^CO precursor, [^11^C]citalopram is a selective serotonin reuptake inhibitor for imaging depressive conditions, which is synthesized by ^11^CH_3_I precursor (82). Various reaction pathways have been shown in Figure 8 to generate secondary precursors using the primary precursors [^11^C]CO_2_ or [^11^C]CH_4_ (83, 84). Three important steps must consider for an effective strategy to synthesize carbon-11 radioligands since it has a shorter half-life, which includes designing a protocol in a way to keep radiolabeling at the final step, the reaction time should be minimal, and reduce isotopic dilution to get a high molar activity. Currently, many organic methodologies have been adapted for developing selective radioligands through C-C bond formation despite using simple methylation with [^11^C]CH_3_I, which include alkylation of nucleophilic groups with carbon-11 inserted alkyl halides, cyanides, and nitroalkanes which can act as electrophilic groups (85), [^11^C]carboxylation reactions with different organometallic copper and other metal-catalyzed reagents, transition metals such as palladium, rhodium, ruthenium, and selenium assisted reactions with different secondary precursors like [^11^C]CH_3_I, [^11^C]CO, and [^11^C]CN (86–88). A few of reported carbon-11 radioligands are synthesized via C-C bond formation include [^11^C]A85380 is a nicotinic Ach receptor agonist, which is synthesized from Pd2(dba)_3_/^11^CH_3_I, [^11^C]MPEP, is a glutamate receptor subtype 5 receptors agonist, which is synthesized from Pd(PPh_3_)_2_Cl_2_/^11^CH_3_I.

## Novel Fluorine-18 and Carbon-11 Radioligands for PET Imaging in Oncology

Gauthier et al. have reported an ^18^F-Labeled Tropomyosin Receptor Kinase (Trk) Inhibitor ^18^F TRACK for PET Imaging. The overexpression of either Tropomyosin Receptor Kinase A, B, or C receptors induces tumor growth, metastasis, and some types of neurological diseases like Alzheimer's and Parkinson's diseases. The ^18^F-TRACK was synthesized *via* Cu mediated radiofluorination reaction in DMF. ^18^F-(R)-TRACK was found to cross the blood-brain barrier (BBB) and showed high brain uptake in studies with non-human primates (89). Zhang et al. have reported ^18^F labeled sulfonamide analog as human carbonic anhydrase IX inhibitor for PET imaging to detect hypoxia tumors. It was synthesized *via a* copper-mediated radiofluorination reaction (90). The studies in the human colorectal cancer xenograft model suggested high uptake in tumors with 0.41 ± 0.06% ID/g. The transmembrane-bound human carbonic anhydrase IX and XII are highly expressed in the tumor cells, and these are considered tumor-associated enzymes, mainly in hypoxic tumors. Therefore, overexpression of human carbonic anhydrase IX and XII are associated with tumor growth, metastasis, and angiogenesis (91–93). Song et al. have reported ^18^F-IRS as Epidermal Growth Factor (EGF) Receptor selective ligand for imaging mutant EGF receptors in non-small-cell lung carcinoma (NSCLC) patients. An overproduction of EGF receptor ligands in the tumor microenvironment leads to cause severe mutations in the EGFR receptors which in turn accelerate epithelial tumor cell growth, invasion, and metastasis (94, 95). This radiotracer showed a good uptake in tumor cells *via* targeting mutant EGF receptors *in vivo*. Wilson et al. have reported ^18^F-Olaparib for PET Imaging of PARP Expression. *In-vitro* studies showed a good tumor uptake of ^18^F -olaparib in PARP-1-expressing Capan-1 and MiaPaCa cells. *In vivo* studies showed specific uptake of ^18^F -olaparib in PARP-1-expressing tumors in mice xenografts model. Overexpression of PARP reduces cellular levels of NAD+, which leads to cause tumor growth, but the exact mechanism is not known (96). Nepal et al. have reported 18F-fluciclovine (Axumin) is a novel radiolabeled amino acid derivative for PET imaging in prostate cancer. This study suggested higher uptake of 18F-fluciclovine in prostate cancer cells. 18F-fluciclovine (Axumin) is the US Food and Drug Administration (FDA) approved radiotracer for PET imaging in patients with recurrent prostate cancer (97). Strebl et al. have reported a new radiotracer [^18^F]MGS3 for PET imaging of histone deacetylase in living subjects, this study confirms high brain uptake, specific binding, and regional distribution of [^18^F]MGS3 (98). Overexpression of histone deacetylases (HDACs) is associated with cancer, neurodegenerative diseases, and psychiatric conditions. Many studies reported various biological targets plays a vital role in cancer progression, one of the major biomarkers is human galectin-1 protein, which is highly expressed in multiple cancers (99, 100), and therefore, which could be the potential targets for novel PET radiotracers to image various conditions in cancer, here a few examples of human galectin-1 selective inhibitors where they have been successfully labeled with fluorine-18 and proved to be PET imaging agents for the diagnosis of cancer (101, 102). The importance of molecular imaging for diagnosis and therapy has been increased in recent days for various cancer types, hence, the current cancer research is spreading widely onto various receptor-based radiotracers.

**Figure 9:**
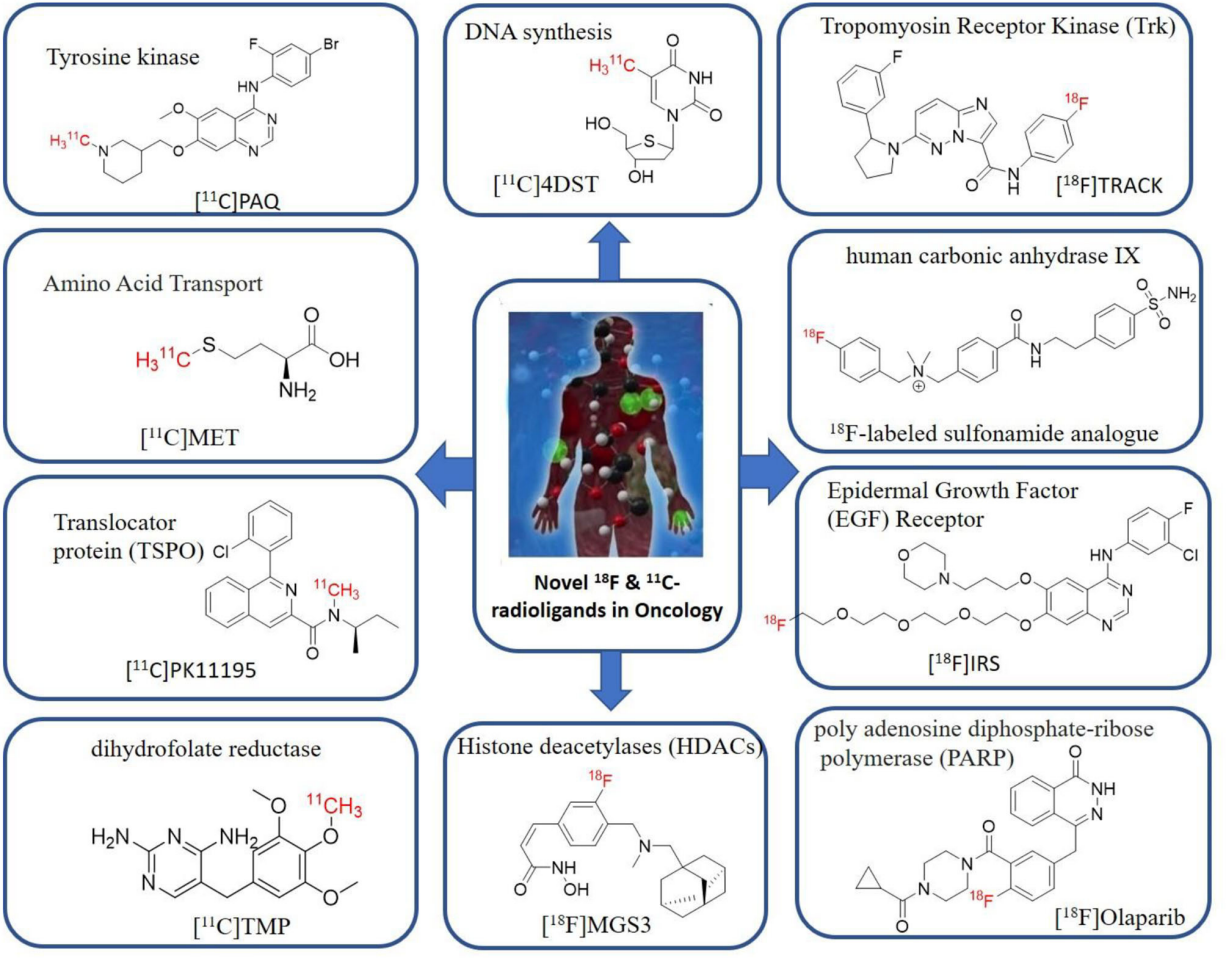
The structures of target-specific novel ^18^F and ^11^C-radiotracers for diagnosis of various conditions in oncology.

Tyrosine kinases are highly upregulated in tumor development, and progression, and are considered cancer hallmarks. Several Tyrosine kinase inhibitors are developed to treat cancer (103). Recently, one study developed carbon-11 labeled 3-Piperidinylethoxyanilinoquinazoline (PAQ), it is a derivative of vandetanib (tyrosine kinase inhibitor), a transgenic mouse model of breast cancer study showed promising results with [^11^C]PAQ to check the cancer treatment status and considered as a good radiotracer in cancer therapy response treatment (104). A thymidine derivative [^11^C]4DST (4′-[methyl-^11^C]thiothymidine) is approved to use clinically for measurement of cancer cells development, metastasis, and invasion. It is one of the hallmarks of cancer cells. The first radiotracer developed was [^11^C]thymidine to measure the proliferative rates in different tumors (105). The large neutral amino acid transporters (LAT) like LAT1, LAT2, LAT3, and LAT4 are highly upregulated in tumor cells due to the full utilization of these transporters for tumor growth and development (106). Various carbon-11 labeled amino acid radiotracers are developed. Recently one study reported an 11C-labeled amino acid radiotracer to quantify tumor cell proliferation (107). Several studies confirmed the upregulation of Translocator protein (TSPO) in gliomas, and a recent study by Su et al. reported the use of [^11^C]PK11195 radiotracer can be easily differentiated between the low-grade astrocytomas and oligodendrogliomas through a PET dynamic study (108). The PET molecular imaging studies with endogenous reporter genes have been translated into clinical research, and one recent study with trimethoprim labeled with carbon-11 ([^11^C]TMP) showed high biodistribution and good sensitivity toward *Escherichia coli* dihydrofolate reductase expressing cells in a xenograft mouse model and suggested it could provide advancement in present PET reporter gene technologies (109). Natural compounds like coumarin and benzimidazole are the most popular ligands for the development of target-based selective anticancer agents, hence which trigger the development of target-based selective ^11^C-radiotracer for molecular imaging in oncology and neurology (110, 111) (Figure 9). Here, we have reported a list of recent patents reported for fluorine-18 and carbon-11 radiotracers with their specific biomarkers for various conditions and clinical use in oncology (Table 4).

**Table 4:** The novel patented ^18^F and ^11^C-radiotracers with their specific biomarkers for various conditions in oncology.

**S. No**	**Radiotracer structure**	**Biological target**	**Applications**
1	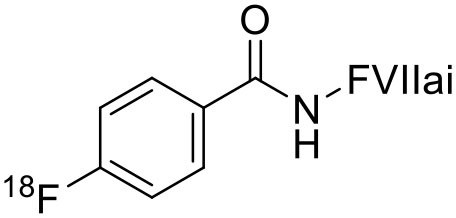 ^18^F-DCFPyL	Prostate-specific membrane antigen (PSMA)	Prostate cancer (112)
2	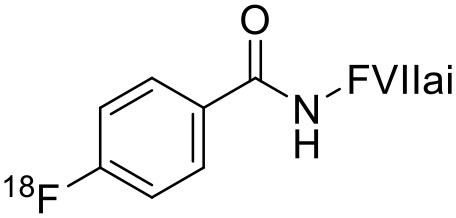 ^18^F-labeled Factor VII PET imaging agent	Tumor tissue factor (TF) with a high binding affinity toward factor VII.	Pancreatic Cancer (113)
3	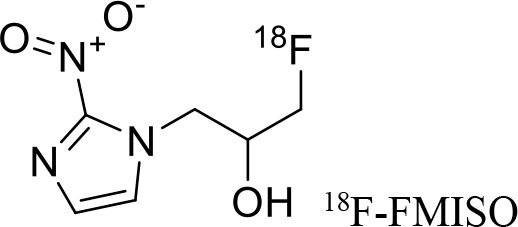 ^18^F-FMISO	Hypoxic cells (macromolecules)	Diffuse Intrinsic Pontine Glioma (114)
4	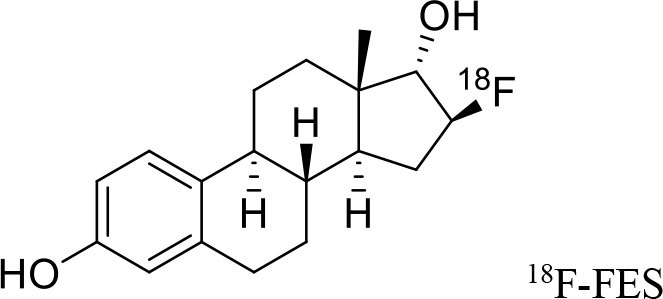 ^18^F-FES	Derivative of estrogen and is used to detect estrogen receptor-positive breast cancer lesions	Breast cancer (115)
5	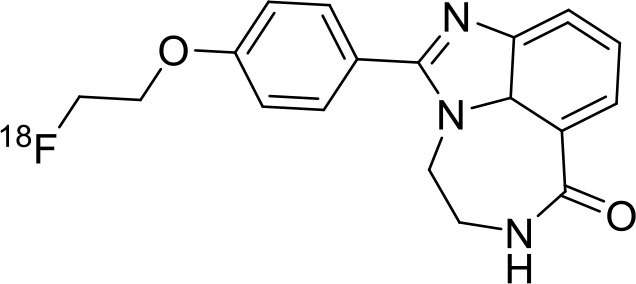 ^18^F-labeled PARP-1 inhibitor	Poly (ADP-Ribose) Polymerase-1 (PARP-1)	Ovarian cancer (116)
6	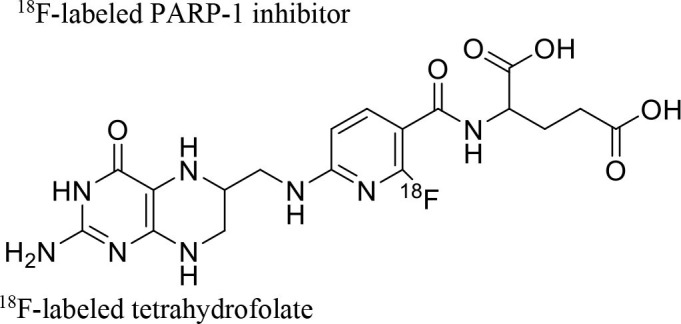 ^18^F-labeled tetrahydrofolate	Folate receptor (FR) expressing cells	Cancer or inflammatory diseases. (117)
7	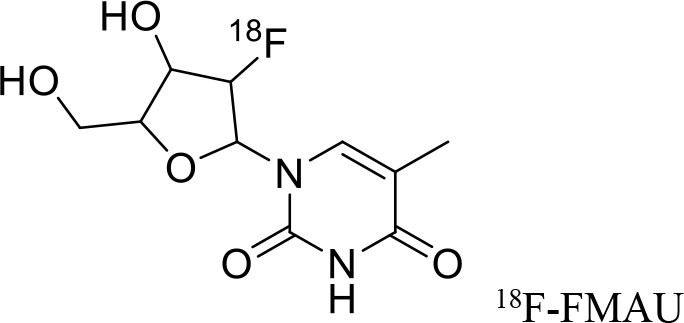 ^18^F-FMAU	It's a thymidine derivative and involved in DNA synthesis.	brain tumors (118)
8	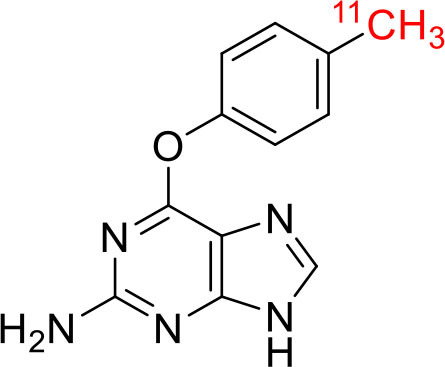 ^11^C-labeled O6 -benzylguanine	O6 -Methyl Guanine Methyltransferase (DNA repair protein)	Malignant brain tumors (119).
9	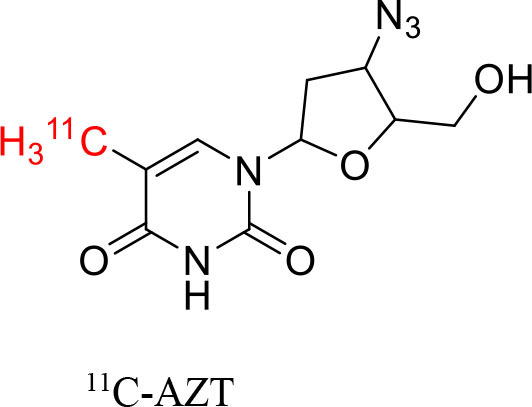 ^11^C-AZT	Nucleoside reverse transcriptase	Glioma tumors (120)
10	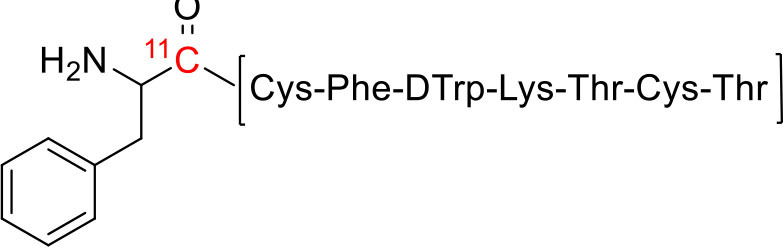 ^11^C-labeled Peptide	Somatostatin receptor	Neuroendocrine tumors (121)

## Novel ^18^F and ^11^C-Radiotracers for PET Imaging in Neurology

A study by Lu et al. reported a new PET ligand [^18^F]LSN3316612 to quantify O-linked-β-N-acetyl-glucosamine (O-GlcNAc) hydrolase (OGA) in the human brain. It enhances the phosphorylation of tau proteins, which are the biomarkers for Alzheimer's disease. Computational and *in-vitro* studies confirmed that LSN3316612 is a selective and potent OGA ligand. Initially, the [^3^H]LSN3316612 was used to image and quantify OGA in the brains of living subjects like rats, monkeys, and humans, and later the [^18^F]LSN3316612 was used to image OGA *in vivo* with the blocking agent thiamet G in monkeys. Furthermore, the research has been extended and reported the PET quantification of O-GlcNAcase in the brain of healthy human volunteers using [^18^F]LSN3316612, the *V*_*T*_ was calculated with two tissue compartment model, and also reported Region-based as well as voxel-based quantification of [^18^F]LSN3316612 in the human brain for O-GlcNAcase (122, 123). Zhang et al. have discovered a novel Radioligand PF-06445974 to image preferably Phosphodiesterase (PDE) 4B-isoform using PET, which is this cold ligand had shown promising results with high potency and selectivity over PDE-4D isoform. The selective PDE-4B inhibitors are used for the treatment of CNS disorders. The Bmax and biodistribution studies confirmed the selectivity of radioligand (124). Wang et al. have clearly reported the detailed structures of all four subtypes of phosphodiesterase-4 leads to providing knowledge to develop selective inhibitors, which could provide the possibility to develop selective subtype phosphodiesterase-4 radioligands (125). Lindberg et al. have developed [^18^F]AZ10419096 radioligand for imaging 5-HT_1B_ receptors. The 5-HT_1B_ receptors abnormalities are associated with the CNS disorders like anxiety, and depression and become a hallmark to study with PET. In a baseline PET study with [^18^F], AZ10419096 showed selectively high brain uptake into 5-HT_1B_ receptor, whereas in blocking PET study with AR-A000002 blocker, the binding was found to be 80% in occipital cortex suggested high specific binding (126). Koole et al. have reported a novel radiotracer [^18^F]MK-6240 for Imaging Neurofibrillary Tangles are a key pathological feature associated with cognitive decline in Alzheimer's disease. The safety of [^18^F]MK-6240 is confirmed by preclinical toxicity studies, first-in-human biodistribution, and dosimetry studies for clinical use in imaging studies of the human brain (127). [^18^F]FPYBF-2, is a novel ^18^F-radiotracer for imaging amyloid plaques in dementia conditions, where this study was performed in 55 dementia patients and 61 healthy volunteers. In Alzheimer's Disease (AD) patients, the ^18^F-FPYBF-2 showed high uptake gray matter and cerebral white suggested as a powerful PET imaging agent for AD (128).

**Figure 10:**
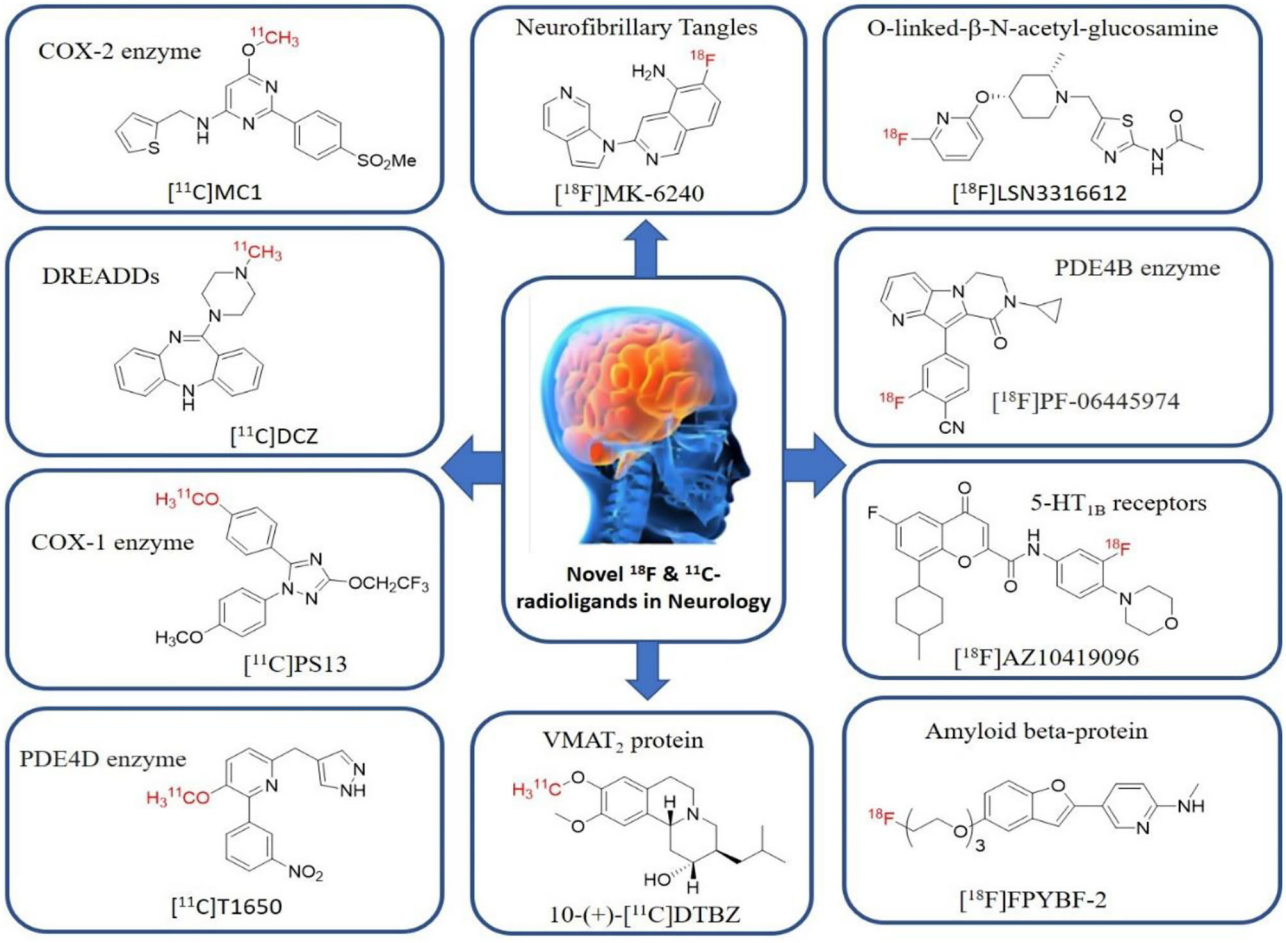
The structures of target-specific novel ^18^F and ^11^C-radiotracers for diagnosis of various conditions in neurology.

Shrestha et al. have reported a novel radioligand [^11^C]MC1 for imaging low-density cyclooxygenase 2 (COX-2) enzyme in the human brain. It is an essential target for neuroinflammation. The COX-1 expression is high in the brain, whereas the COX-2 expression is low in healthy brain tissue, but it is upregulated during the inflammatory process. This [^11^C]MC1 is a selective radioligand for COX-2 and found that in rheumatoid arthritis conditions it detected high-density COX-2 in symptomatic joints. This study shows high uptake of [^11^C]MC1 in the human brain and the specific binding to COX-2 was confirmed by blocking studies with celecoxib (129). Yan et al. have reported an improved PET radioligand [^11^C]deschloroclozapine ([^11^C]DCZ) to quantify the muscarinic DREADDs transfected in the brain of monkeys. In the monkey DREADDs model, the signal-to-background ratio of [^11^C]DCZ was almost 2-fold greater than that of [^11^C]clozapine ([^11^C]CLZ) due to a much lower background uptake. Hence, it is suggested that the [^11^C]deschloroclozapine has high selectivity over DREADD hM4Di receptors than [^11^C]CLZ (130). The Designer Receptors Exclusively Activated by Designer Drugs (DREADDs) are a novel chemogenetic technology used to activate or inhibit different neural populations in the brain regions leading to control various neurological conditions (131). Kim et al. have reported the first-in-human evaluation of a novel radioligand [^11^C]PS13 to quantify cyclooxygenase-1 (COX-1) in the brain. It is also an essential target for neuroinflammation. The pharmacokinetic profiles were obtained through two-tissue compartment models, and Logan graphical analysis, the total distribution volume (V_T_) was also determined. This study found that [^11^C] PS13 shows high uptake in the occipital cortex and hippocampal regions of the human brain (132). Wakabayashi et al. have reported a selective phosphodiesterase subtype 4D (PDE4D) radioligand, it is a potential target to improve cognition or antidepression category. This study has reported four lead compounds having an alkoxypyridinyl base with high *in vivo* PDE4D selectivity, potency, lipophilicity, and good brain uptake. The blocking studies with rolipram or BPN14770 (selective PDE4D inhibitor) confirmed specific binding of [^11^C]T1650 in the cognitive functions associated regions like the prefrontal cortex, hippocampus, and temporal cortex, and the major limitation of this study is the high brain enzyme density (*V*_T_) with scan duration because of accumulation of radiometabolites in the brain (133). The vesicular monoamine transporter 2 (VMAT_2_) protein is considered a biomarker to evaluate the condition of Parkinson's disease. The synthesized dopamine at the striatal cells is transported and stored in the synaptic storage vesicles mediated by VMAT_2_ receptors. Hence, the recent study developed a novel radiotracer 10-(+)-[^11^C]DTBZ as *in vivo* PET imaging for VMAT_2_ using micro-PET study to understand Parkinson's disease condition (134) (Figure 10). Here, we have reported a list of recent patents reported for fluorine-18 and carbon-11 radiotracers with their specific biomarkers for various conditions and clinical use in neurology (Table 5).

**Table 5:** The novel patented ^18^F and ^11^C-radiotracers with their specific biomarkers for various conditions in neurology.

**S. No**	**Radiotracer structure**	**Biological target**	**Applications**
1	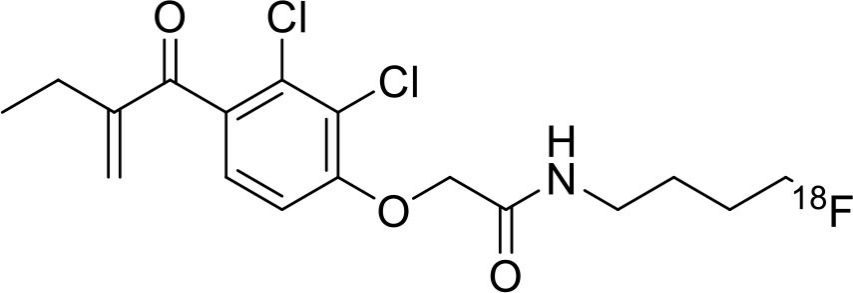 ^18^F-labeled fluorobutyl ethacrynic amide	Lipocalin-type prostaglandin D synthase (L-PGDS) enzyme	Alzheimer's disease (135)
2	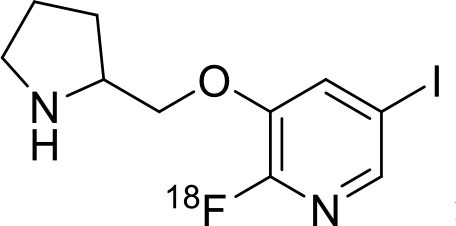 ^18^F-labeled nicotine	Nicotinic acetylcholine receptor (nAChR) Agonist	Parkinson's disease (136)
3	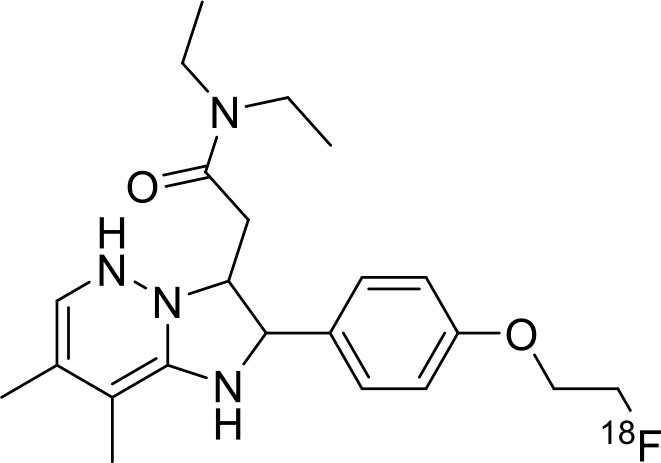 ^18^F-labeled imidazo [1,2-b] pyridazine derivative	Translocator protein (TSPO).	Neuroinflammation in neurological conditions (137)
4	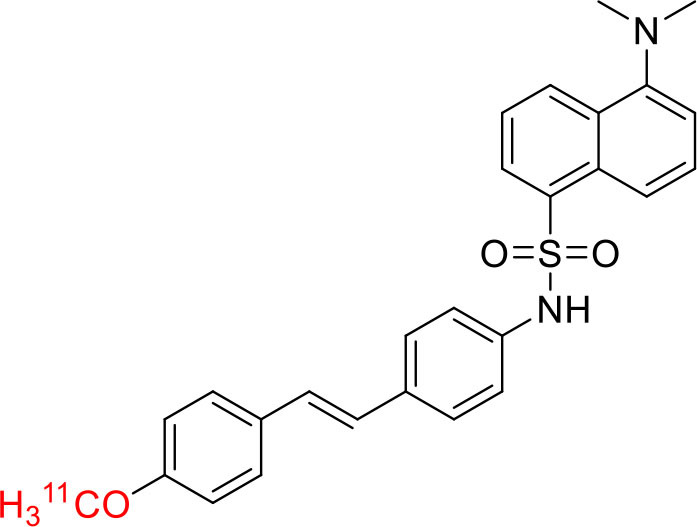 ^11^C-DSB	β-amyloid (Aβ) plaques	Alzheimer's disease (AD) (138)
5	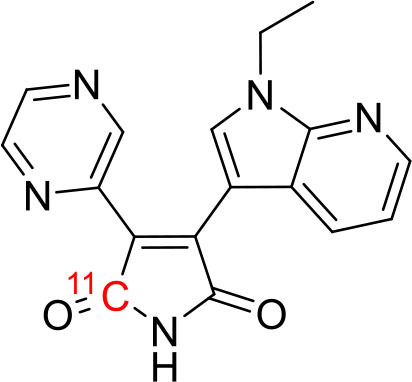 ^11^C-Pyrrolidine-2,5-dione analog	Glycogen Synthase Kinase-3	Senile dementia (139)
6	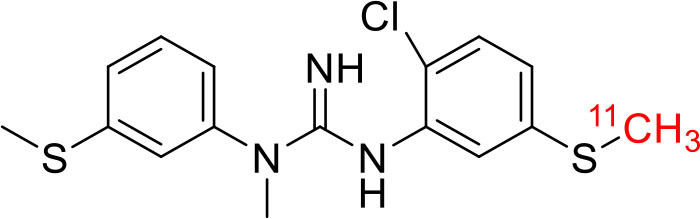 ^11^C-phenyl-N'-methylguanidine analog	N-methyl-D-aspartate (NMDA) receptor	Alzheimer's disease, and Huntington's disease (140)
7	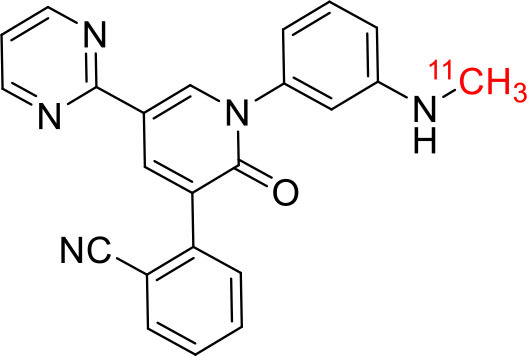 ^11^C-pyrimidine analog	Glutamate receptors (mGluRs)	Parkinson's disease, neuropathic pain (141)
8	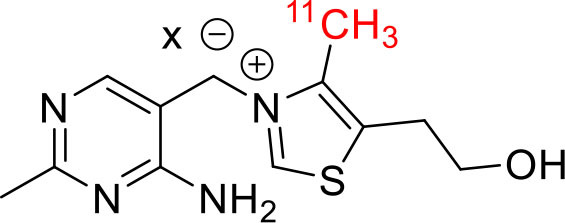 ^11^C-fursultiamine analog	Octamer-binding transcription factor 4 (OCT-4)	Abrogate resistance to chemo- and radiotherapy (142)
9	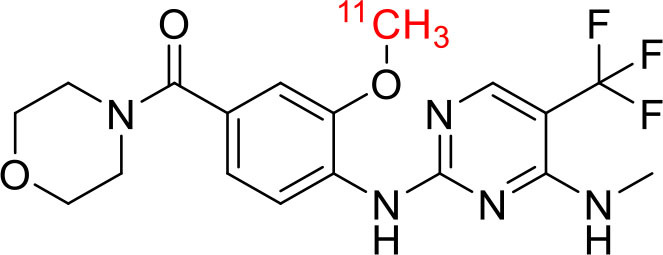 ^11^C-G1023	Leucine-rich repeat kinase 2 protein (LRRK2)	Parkinson's disease (143)
10	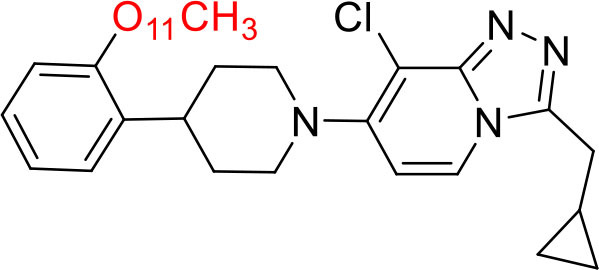 ^11^C-triazolo [4,3 -a]pyridine analog	Glutamate receptor mGluR2	Schizophrenia, anxiety, depression (144)

## Future Perspectives

The PET is the best technology with great potential and limited challenges in the field of molecular imaging. It plays a tremendous role in the medical diagnosis of various conditions in oncology, cardiology, and neurology. The specialty of PET is to measure lower concentrations like 10^−6^-10^−9^ gm of radiotracers in living subjects. The drawback of PET is to provide limited anatomic data which is overcome by hybrid technology such as PET-CT, and PET-MR for attenuation correction and anatomical orientation. The PET is more sensitive (10^−12^ M) than other medical imaging modalities like MRI (10^−4^ M), but it has a low special resolution (2–6 mm). Therefore, PET remains a remarkable molecular imaging modality in biomedical as well as clinical practice. Micro dosing is one of the best approaches in pharmaceutical development since the low concentration of radiopharmaceutical exhibits lower toxicologic risks. The total-body PET/CT system, like a whole-body dynamic scan with high sensitivity, makes it useful for radiolabeled drug pharmacokinetic study. Therefore, the distribution of radiopharmaceuticals can be visualized, and the time of activity can be calculated in different tissue regions of interest simultaneously with dynamic images. Hence, pharmacokinetics, as well as pharmacodynamics, can be estimated more precisely. The total-body PET/CT has many advantages in the evaluation of various systemic diseases, which include higher sensitivity allows imaging at high signal to noise ratio (SNR) at later time points after radioligand injection, the total-body dynamic scan provides more kinetic information than normal conventional PET/CT, and the whole-body images can provide more comprehensive evidence of many of systemic diseases in oncology, cardiology, and neurology (145, 146). The fluorine-18 and carbon-11 are promising radiotracers with many advantages but limited challenges. The fluorine-18 can withhold long-run radiochemical synthesis due to a longer half-life of 110 min, and multiple studies can be done by PET in a single day. Despite the shorter half-life of carbon-11 (*t*_1/2_ = 20.4 min), it is widely used in various synthetic methodologies with different heterocycles because of the direct insertion of the carbon-11 radionuclide. Several automated methods are adapted for easy, rapid, and efficient synthesis of various PET radiopharmaceuticals. Therefore, advances in multimodality devices could help in getting accurate and reliable data on drug candidates at different stages of drug development.

## Conclusions

In this article, we have focused on the importance of PET molecular imaging in the drug discovery and development process. The PET is mainly used to address essential factors to advance the drug development process, which include understanding the pathophysiology, measuring roles and applications of PET molecular imaging in drug discovery and development. In addition, it is also focused on a variety of carbon-11 and fluorine-18 radioligands, their modified and advanced procedures of production, and their PET imaging applications in early state diagnosis, prognosis, and treatment of disease conditions in oncology, and neurology. Various radiofluorination methods, easy synthesis, appropriate precursor identification, and automated synthesis measures have been explored to reach the goals of developing selective radiotracers. Clinical use of PET imaging agents with carbon-11 and fluorine-18 have vastly increased due to a wide range of advantages. It would be helpful to understand the significance of PET molecular imaging in drug development, and the importance of positrons emitting fluorine-18 and carbon-11 radiotracers.

## Author Contributions

SN and PS: data collection, writing, analyzing, and setting up paper. TS: data collection, analysis, and review. CD: editorial role to the paper. All authors contributed to the article and approved the submitted version.

## Conflict of Interest

The authors declare that the research was conducted in the absence of any commercial or financial relationships that could be construed as a potential conflict of interest.

## Publisher's Note

All claims expressed in this article are solely those of the authors and do not necessarily represent those of their affiliated organizations, or those of the publisher, the editors and the reviewers. Any product that may be evaluated in this article, or claim that may be made by its manufacturer, is not guaranteed or endorsed by the publisher.
